# Essential procedures of single-cell RNA sequencing in multiple myeloma and its translational value

**DOI:** 10.1097/BS9.0000000000000172

**Published:** 2023-11-02

**Authors:** Jun Du, Xiao-Ran Gu, Xiao-Xiao Yu, Yang-Jia Cao, Jian Hou

**Affiliations:** a Department of Hematology, Renji Hospital, School of Medicine, Shanghai Jiao Tong University, Shanghai 200127, China; b School of Medicine, Shanghai Jiao Tong University, Shanghai 200025, China; c Department of Hematology, First Affiliated Hospital of Xi’an Jiaotong University, Xi’an, Shanxi 710000, China

**Keywords:** Evolution, Microenvironment, Multiple myeloma, scRNA-seq, Target therapy

## Abstract

Multiple myeloma (MM) is a malignant neoplasm characterized by clonal proliferation of abnormal plasma cells. In many countries, it ranks as the second most prevalent malignant neoplasm of the hematopoietic system. Although treatment methods for MM have been continuously improved and the survival of patients has been dramatically prolonged, MM remains an incurable disease with a high probability of recurrence. As such, there are still many challenges to be addressed. One promising approach is single-cell RNA sequencing (scRNA-seq), which can elucidate the transcriptome heterogeneity of individual cells and reveal previously unknown cell types or states in complex tissues. In this review, we outlined the experimental workflow of scRNA-seq in MM, listed some commonly used scRNA-seq platforms and analytical tools. In addition, with the advent of scRNA-seq, many studies have made new progress in the key molecular mechanisms during MM clonal evolution, cell interactions and molecular regulation in the microenvironment, and drug resistance mechanisms in target therapy. We summarized the main findings and sequencing platforms for applying scRNA-seq to MM research and proposed broad directions for targeted therapies based on these findings.

## 1. BACKGROUND

Multiple myeloma (MM) is a heterogeneous malignant plasma cell disorder originating from bone marrow (BM) plasma cells.^1^ MM evolves most commonly from an asymptomatic precursor condition, known as monoclonal gammopathy of undetermined significance (MGUS), which progresses to smoldering MM (SMM) and ultimately evolves into relapsed or refractory MM (RRMM).^2–4^ Unfortunately, despite advances in treatment, MM remains incurable. Intratumor heterogeneity significantly challenges effective tumor response, prognosis, and survival.^5^ The traditional bulk-tissue analysis only provides a virtual average of the multiple cellular components. The conventional analysis of bulk tissue only provides an average representation of multiple cellular components. However, the emergence of single-cell RNA sequencing (scRNA-seq) technologies has enabled a comprehensive and unbiased examination of gene expression patterns and cell fate decisions at high resolution and depth for individual cells.^6,7^ For this reason, scRNA-seq has played a profound role in investigating the pathogenesis of MM and guiding clinical treatment.

In this review, we provide a comprehensive overview of the strengths and limitations of various single-cell sequencing platforms and analytical tools as well as delineate the experimental workflow for scRNA-seq in MM. Furthermore, we summarize the research progress using scRNA-seq that has substantially improved our experience with single-cell sequencing technologies in disease research and has also helped to improve our understanding of the underlying mechanisms of MM.

## 2. SINGLE-CELL RNA SEQUENCING TECHNIQUES

**Figure 1.**
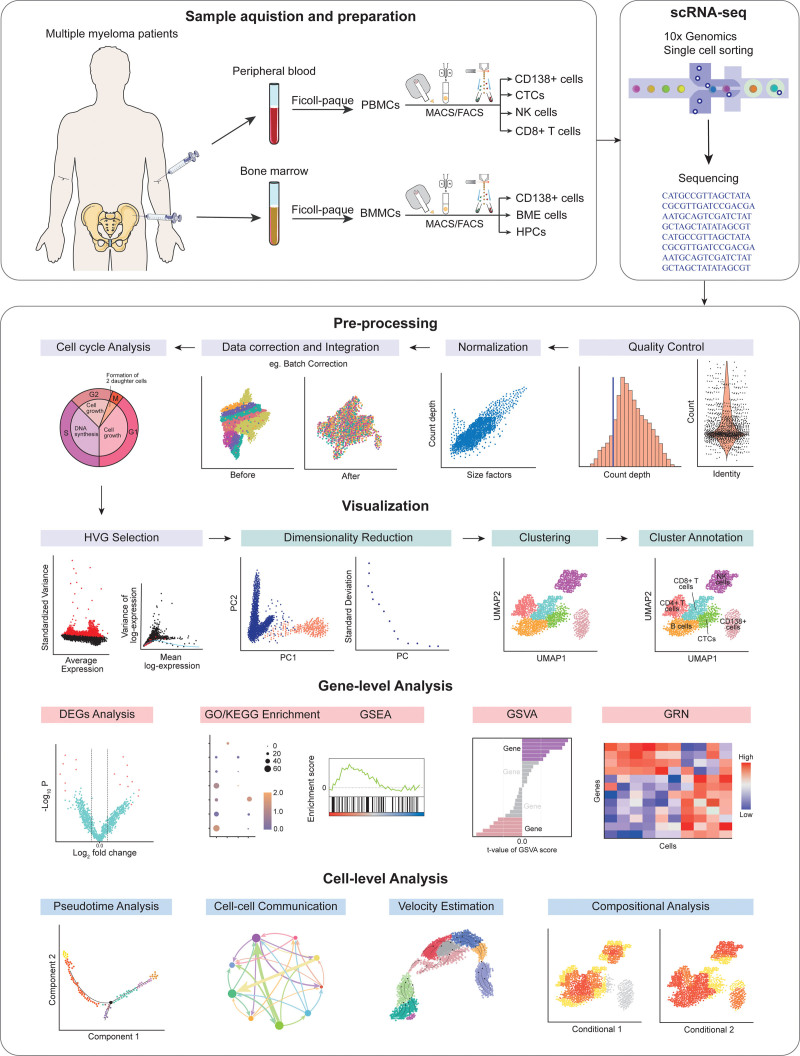
Schematic workflow of scRNA-seq data creation and analysis. BMMC = bone marrow mononuclear cell, DEG = differentially expressed genes, GO = Gene Ontology, GRN = gene regulatory network, GSEA = gene set enrichment analysis, GSVA = gene set variation analysis, HVG = highly variable gene, KEGG = Kyoto Encyclopedia of Genes and Genomes, PC = principal component, PBMC = peripheral blood mononuclear cell, UMAP = uniform manifold approximation and projection.

The process of single-cell RNA sequencing (scRNA-seq) involves a series of steps, which have been summarized in Figure 1.

### 2.1. Samples acquisition and preparation

Unlike several solid tumor samples^8,9^ that require mechanical dissociation and enzymatic digestion, MM is a tumor of the BM and blood system, and samples obtained from the peripheral blood (PB) and BM aspirates do not require much complicated processing to produce cell suspensions. After that, the cell type required for sequencing is separated from mononuclear cells obtained by density gradient centrifugation using magnetic activated cell sorting (MACS)^10^ or fluorescence-activated cell sorting.^11^ In conjunction with light scatter characteristics, the combination of CD38, CD138, and CD45 markers has emerged as the preferred approach for identifying and quantifying plasma cells. Furthermore, the utilization of CD19, CD56, CD117, CD20, CD28, CD27, and CD81 expressions alongside CyIg light-chain restriction is associated with distinct behavioral patterns that enable unequivocal differentiation between clonal and normal/reactive plasma cells.^12^

### 2.2. Principle scRNA-seq pipeline

Once a certain concentration of cell suspension is obtained, various scRNA-seq protocols are available for sequencing. For scRNA-seq protocols, the key difference is whether the sequencing is a full-length transcript or 5′/3′-end fragment. Additionally, depending on the different cell capture strategies employed, scRNA-seq protocols can be categorized as either droplet-based or plate-based. Protocols with low cell capture efficiency may only be reliable for analyzing modest to abundant cell components.^13^ Protocols for RNA sequencing are briefly summarized in Table 1. Researchers can choose different protocols according to their research requirements. After that, the following steps are typically involved: capture mRNA from single-cell lysis; reverse transcribing the mRNA into cDNA and amplifying it through polymerase chain reaction (PCR) or in vitro transcription; library construction; and sequencing. To begin analyzing scRNA-seq data, the first step is to process the raw reads into feature-barcode matrices that can be used in downstream analyses. If the 10× genomics protocol^14^ was used for library construction, CellRanger offers a convenient method for this processing, albeit slow and memory intensive. Alternative read processing approaches such as DropEst, Kallisto-BUStools, UMI-Tools, STARSolo, and Alevin are optimized for runtime and memory, allowing users to process their scRNA-seq runs without investing as much in computational infrastructure.^29^ Some downstream analytical tools are summarized in Table 2.

**Table 1.** A brief overview of scRNA-seq library construction protocols.

Platforms	Throughput (cells per run)	Sequencing depth	Cell isolation strategies	Library construction	UMI	Advantage	Limitation	Commercial platform	Ref
Chromium	>10,000	10^4^–10^5^ reads per cell or 4–5 × 10^3^ genes per cell	Droplet-based	3′/5′-fragments or full-length (through TSO)	Yes	High throughput; diverse sample compatibility; cell size flexibility; rapid encapsulation of single cell; simple to operate	High requirement of initial cell concentration and cell activity	10× Genomics	^14–16^
SPLiT-seq	>100,000	5 × 10^5^ reads per cell or 4–5 × 10^3^ genes per cell	None	3′-fragments	Yes	High throughput; low cost; simple equipment requirements; a multistep barcoding strategy	Only 3′-fragments instead of full-length	/	^17^
inDrop	>10,000	10^5^–10^6^ reads per cell or 5–15 × 10^3^ genes per cell	Droplet-based	3′-fragments	Yes	High throughput; an open-source system; easily modified with different protocols; large cross-section channel without cell size bias in capture	Low cell capturing efficiency (~7%); no analysis software support; skills to operate required	/	^13,15^
Drop-seq	>10,000	10^5^–10^6^ reads per cell or 5–6 × 10^3^ genes per cell	Droplet-based	3′-fragments	Yes	High throughput; open configurable system to develop new protocols and applications	Low cell capturing efficiency (10%–20%); less suited for rare cells; low sensitivity of single-cell genes	/	^15,18^
ddSEQ	>10,000	10^3^–10^4^ reads per cell	Droplet-based	3′-fragments	Yes	Agnostic to cell size; sensitive and unbiased characterization of chromatin accessibility or transcriptional signatures	No user’s modification possible; extremely low cell capture rate	Bio-Rad Illumina	^19^
CEL-seq2	100–1000	10^3^–10^4^ genes per cell	Plate-based (Micropipette)	3′-fragments	Yes	More time- and cost-efficient and accuracy than CEL-seq	Micropipette isolation strategy make it still difficult and time-consuming	/	^20^
SMARTer	96/384	10^5^–10^7^ reads per cell or 4–8 × 10^3^ genes per cell	Microfluidics	Full-length	No	End-to-end solution; available for deep and shallow sequence; allows cells to be individually imaged prior to sequencing	Work for cells relatively homogeneous in size; low capture efficiency for sticky and nonspherical cells	Fluidigm C1	^21,22^
MATQ-seq	10–100	10^4^ genes per cell	Plate-based (mouth pipette)	Full-length	Yes	Can detect both polyA+ and polyA- RNAs; high sensitivity and accuracy	High cost, high technical requirement and time-consuming	/	^23^
SUPeR-seq	100–1000	10^4^ genes per cell	Plate-based (mouth pipette)	Full-length	No	Can detect both polyA+ and polyA- RNAs such as circRNAs; uses random primers instead of oligo (dT) primers	Contains about 15% rRNA of the overall cDNA; mild 3′-end bias	/	^24^
CytoSeq	100 to >10,000	8–9 × 10^3^ genes per cell	Plate-based (microwell array)	3′-fragments	Yes	High throughput; low cost; capture and analyzation of fragile cells; no restriction for cell size and shape	High requirement of initial cell number; process is complex	BD Rhapsody	^25,26^
Smart-seq2	100–1000	10^4^ genes or 10^5^–10^6^ reads per cell	Plate-based (Micropipette)	Full-length	No	Can detect genes at low expression levels; no requirement for previous bead purification	Applies only to polyadenylate RNA, no strand or molecular information; high cost, high technical requirement and time-consuming; mild 5′-end bias	/	^16,27,28^

**Table 2.** Tools for single-cell sequencing data analysis.

Tools	Year	Applications	Descriptions	Software	Ref
Preprocessing					
Scanpy	2018	Toolboxes for scRNA-seq data analysis	Python package; workflow is modular	https://github.com/theislab/Scanpy	^30^
Scater	2017	Toolboxes for scRNA-seq data analysis	R package	http://bioconductor.org/packages/scater	^31^
Granatum	2017	Toolboxes for scRNA-seq data analysis	Web package; an easy-to-use graphical interface without programming code	http://garmiregroup.org/granatum/app	^32^
ASAP	2017	Toolboxes for scRNA-seq data analysis	Web, R and Python package; easy to use; short run time	https://asap.epfl.ch/, https://github.com/DeplanckeLab/ASAP	^33^
Scran	2016	Toolboxes for scRNA-seq data analysis	R package; workflow is modular; normalizes data using pooling-based size factor estimation and linear regression	https://bioconductor.org/packages/release/bioc/html/scran.html	^34^
Seurat	2015	Toolboxes for scRNA-seq data analysis	R package; can jointly analyze multiple data sets with overlapping and non-overlapping populations	https://github.com/satijalab/seurat	^35,36^
SINCERA	2015	Toolboxes for scRNA-seq data analysis	R package	https://research.cchmc.org/pbge/sincera.html	^37^
popsicleR	2022	Quality control, filter low-quality cells, normalization, remove technical and biological biases, cell clustering and annotation	R package; can be applied at multiple resolutions; can return dot plots showing	https://github.com/bicciatolab/popsicleR	^38^
miQC	2021	Quality control	R package; identifies high-quality cells within an individual sample; adaptive across experimental platforms	https://bioconductor.org/packages/miQC	^39^
RSeQC	2012	Quality control	R package; includes basic modules and RNA-seq specific modules	http://code.google.com/p/rseqc/	^40^
scds	2020	Doublet removal	R and python package; short run time; can detect doublets in scRNA-seq data	https://github.com/kostkalab/scds_manuscript	^41^
Scrublet	2019	Doublet removal	Python package; identify technological doublets generated by random cell co-encapsulation	https://github.com/AllonKleinLab/scrublet	^42^
SCTransform	2019	Normalization	R package; for UMI-based scRNA-seq dataset; the results can eliminate the effect of sequencing depth	https://github.com/satijalab/sctransform	^43,44^
SCnorm	2017	Normalization	R package; grouping genes with comparable dependence on total UMI or read count and determining size factors within each pool	http://www.biostat.wisc.edu/~kendzior/SCNORM/	^45^
fastMNN	2018	Batch correction	R package; there must be at least one shared cell population between batches; using a Gaussian kernel to adjust for local variations	https://bioconductor.org/packages/scran/	^46^
LIGER	2019	Batch correction	R package; uses integral nonnegative matrix factorization; retains the basic cell-type structure in the integrated space better than s software	https://github.com/MacoskoLab/liger	^47^
CCA	2018	Batch correction	R package; efficiently discover conserved gene connection patterns and common biological indicators; not affected by linear changes in gene expression; is paired PCA; implemented in the R toolkit Seurat	http://satijalab.org/seurat/	^36,48^
Harmony	2019	Cell clustering, trajectory analysis, visualization, integrates dissociated scRNA-seq with spatially resolved datasets	R package; projects cells into a shared embedding; cells group by cell type	https://github.com/immunogenomics/harmony	^49^
Visualization					
PHATE	2019	Nonlinear dimensionality reduction, clustering	R, Python package and MATLAB program; captures both local and global nonlinear structure; embeds distance information into 2 or 3 dimensions	https://github.com/KrishnaswamyLab/ PHATE	^50^
UMAP	2018	Nonlinear dimensionality reduction	Python package; computes outputs fast; can capture global structure in discrete datasets	https://github.com/lmcinnes/umap	^51,52^
scvis	2018	Dimensionality reduction	Python package; neural network is used to learn parametric mapping from high- to low- dimensional space; provides log-likelihoods	https://bitbucket.org/jerry00/scvis-dev	^53^
SWNE	2018	Dimensionality reduction	R package; uses nonnegative matrix factorization; captures both local and global structure	https://github.com/yanwu2014/swne	^54^
SIMLR	2017	Multikernel learning for dimensionality reduction, clustering and visualization	R package and MATLAB program; unsupervised methods; can solve high levels of dropout events	https://github.com/BatzoglouLabSU/SIMLR	^55^
Cytosplore^+HSNE^	2017	Nonlinear dimensionality reduction, clustering	C + +, Javascript and OpenGL program; short run time; can identify rare cell populations missed due to downsampling	https://github.com/Nicola17/High-Dimensional-Inspector	^56^
ZIFA	2015	Zero-inflated and linear dimensionality reduction	Python package; values near zero can be interpreted in a univariate hybrid modeling framework	https://github.com/epierson9/ZIFA	^57^
ACCENSE	2014	Nonlinear dimensionality reduction	R package and MATLAB program; combines t-SNE with density-based partitioning	http://www.cellaccense.com	^58^
viSNE	2013	Dimensionality reduction	A fast, distributed implementation of t-SNE; includes cyt; implementation available in Cytobank	http://www.c2b2.columbia.edu/danapeerlab/html/cyt.html	^59^
SPADE	2011	Dimensionality reduction, clustering and annotation	R package; an unsupervised manner; visualization of cell types in a branch tree structure	https://github.com/nolanlab/ spade	^60^
t-SNE	2008	Nonlinear dimensionality reduction	Several implementations available; can be implemented via Barnes-Hut approximations	https://lvdmaaten.github.io/tsne/	^61^
SC3	2017	Unsupervised clustering	R package; complete-linkage hierarchical clustering	http://bioconductor.org/packages/SC3	^62^
FlowSOM	2015	Clustering	R package; clustering can be visualized in a minimal spanning tree; visualization of several markers by star charts or cell types by pie charts	https://github.com/SofieVG/FlowSOM	^63^
singleR	2019	Cluster annotation	R package; uses reference transcriptome datasets of pure cell types to infer the cell of origin of each individual single cell	https://bioconductor.org/packages/release/bioc/html/SingleR.html	^64^
Gene-level analysis					
GO		Source of information on the functions of genes	A database; knowledge is both human- and machine-readable	http://geneontology.org	
KEGG		Bioinformatics resource for deciphering the genome	A database; is utilized for bioinformatics research; includes all organisms	https://www.genome.jp/kegg/	
DESeq2	2014	Differential expression analysis	R package; uses shrinkage estimation for dispersions and fold changes	https://github.com/mikelove/DESeq2	^65^
edgeR	2010	Differential expression analysis	R package; examining differential expression of replicated count data	https://bioconductor.org/packages/release/bioc/html/edgeR.html	^66^
GSEA	2005	Gene set enrichment analysis	Java-based; identifies classes of genes or proteins that are overrepresented	https://github.com/GSEA-MSigDB/gsea-desktop	^67^
GSVA	2013	Gene set variation analysis	R package; a nonparametric and unsupervised method; estimates variation of gene set enrichment	https://github.com/rcastelo/GSVA	^68^
SCENIC	2017	Gene regulatory network analysis	R and Python package;	https://scenic.aertslab.org/	^69^
Cell-level analysis					
Monocle3	2019	Trajectory analysis; clustering; differential expression analysis	R package; can track changes over pseudo time; analyses how cells choose between one of several possible end states	https://cole-trapnell-lab.github.io/monocle3/	^70^
PAGA	2019	Trajectory analysis; clustering	Jupyter Notebook; available with scanpy; preserves the global topology of data	https://github.com/theislab/paga	^71^
Destiny	2016	Trajectory analysis, nonlinear dimensionality reduction	R package; uses diffusion maps	https://github.com/theislab/destiny/	^72^
Connectome	2019	Explore cell-cell connectivity patterns	R package; work with Seurat; no spatial registration	https://msraredon.github.io/Connectome/	^73^
CellPhone DB	2018	Repository of ligand–receptor interaction	Python package; accurately represents heteromeric complexes; includes existing and new manually curated information	https://www.cellphonedb.org/ https://github.com/Teichlab/cellphonedb	^74^
ScVelo	2020	Velocity estimation	Python package; uses a likelihood-based dynamical model to solve the full transcriptional dynamics of splicing kinetics	https://scvelo.readthedocs.io/	^75^
Velocyto	2018	Velocity estimation	R and Python package; can predict the future state of individual cells	http://velocyto.org/	^76^
scCODA	2021	Compositional analysis	Jupyter Notebook and Python package; a Bayesian model; identifies cell-type change	https://github.com/theislab/scCODA	^77^

GO = Gene Oncology, KEGG = Kyoto Encyclopedia of Genes and Genomes.

### 2.3. Quality control

Data quality can be impacted by technical artifacts associated with cell dissociation, cell encapsulation, library preparation, or sequencing processes.^78^ Cells that exhibit relatively small library sizes, low gene expression levels, or a high proportion of reads or unique molecular identifiers mapping to mitochondrial DNA (mtDNA)-encoded genes are considered to be of low quality.^34^ Prior to downstream analysis, it is necessary to eliminate low-quality cells and systematically evaluate technical artifacts.^79^ Most scRNA-seq protocols may encapsulate 2 or more cells and barcode in 1 reaction volume, and the data that represent multiplets can use doublet removal^42^ package to remove. Existing tools such as miQC,^39^ Scanpy,^30^ Seurat,^35^ and Scater^31^ can be used to filter cells based on user-defined thresholds of these parameters.

### 2.4. Normalization and data correction and Integration

Normalization is the process of removing technical effects typically arise from cDNA capture or PCR amplification efficiency across cells while preserving true biological variation such as heterogeneity or differential expression.^43^ The normalization of scRNA-seq can be roughly divided into two broad categories. One is based on “size factors,” such as BASICS corrected with spike-ins,^80^ Scran,^34^ which measures size factors with multiple “cell pools,” and TMM^81^ and DESeq,^82^ which are traditionally used for bulk transcriptional standardization. The other is a probability distribution-based approach that fits specific parameters according to the expression distribution of each gene and then normalizes each gene, such as SCnorms,^45^ SCTransform,^43^ and ZINB adopted by scVI.^83^ A normalized measure is then used for downstream analysis, such as the detection of highly variable features, clustering and differential expression.^84^ Other detailed normalization methods comparison have been published previously.^43,85^

Despite normalization, unintended variabilities such as batch, dropout, or cell cycle effects^86^ may still occur. Many studies incorporate sequencing data from multiple experiments. These data generated using different reagents, handling protocols, equipment, or sequencing platforms result in batch effects.^46^ To date, the batch-effect removal methods have advantages and limitations, with no method clearly superior. Harmony,^49^ however, performed better when analyzing large datasets and common cell types.^87^ Moreover, data correction arising from cell cycle effects can be corrected by performing a simple linear regression on cell cycle scores, implemented in both Scanpy^30^ and Seurat^35^ platforms.

### 2.5. Highly variable genes selection and dimensionality reduction

With a large number of cells being measured, single-cell datasets tend to have high dimensions and introduce noise and complex calculations into computational work. In homogeneous cell populations, highly variable genes (HVGs) are detected to identify genes with the strongest biological signal that significantly contribute to cell-to-cell variability. Some functions like “trendVar” and “decomposeVar” from the scran,^34^ “FindVariableFeatures” from the Seurat,^35^ “BrenneckeGetVariableGenes” from the M3Drop^88^ can be used to select HVGs. Restricting downstream analyses to the most informative genes can mitigate the impact of dimensionality and noise, while simplifying the analysis.^89^ To further reduce computational complexity and negative effects associated with high-dimensional expression matrices, dimensionality reduction algorithms employing both linear and nonlinear methods have been developed. A comparison of various dimensionality reduction techniques has previously been published.^90^ The most commonly employed linear transformation strategies involves principal component analysis (PCA), which identifies axes (or PC) that capture the greatest amount of variation in high-dimensional space. Moreover, since PCA preserves meaningful information in early axes, the number of PCs selected for subsequent analysis affects the magnitude of relevant information loss or noise, which results in distortion of the basic pattern of variation/covariation.^91^

Unlike PCA, nonlinear dimensionality reduction algorithms such as t-distributed stochastic neighbor embedding (t-SNE)^61^ and uniform manifold approximation and projection (UMAP)^51^ are not confined to linear transformation, offering greater flexibility in arranging cells in low-dimensional space. This allows for the separation of unique clusters within complex populations. Furthermore, UMAP is increasingly supplanting t-SNE as the preeminent method for dimensionality reduction due to its fast computation time, capacity to meaningfully represent extremely large datasets and retention of large-scale information.^51^

### 2.6. Unsupervised clustering and cell-type annotation

Unsupervised clustering is a critical step in the visualization process of scRNA-seq data analysis, enabling identification of cell clusters with similar expression profiles. These clusters may represent distinct cell types or different stages of the same type. Unlike supervised clustering, which relies on predefined labels for cells, unsupervised clustering focuses solely on the data and employs algorithms to group cells. A variety of clustering methods have been developed for scRNA-seq analysis, including the widely used k-means algorithm based on minimizing the sum of squared Euclidean distances,^92^ hierarchical clustering based on constructed branch of cell types and subtypes,^93^ density-based clustering based on the density of datapoints in the input space,^94^ and graph-based clustering assuming that dense communities in a graph can be represented as spectral components or dense subsets.^95^ Apart from these, there are also clustering algorithms such as mixture models,^96^ neural networks,^97^ ensemble clustering,^62^ and affinity propagation.^98^ Their strengths, limitations, and time complexity are summarized in this article.^99,100^

Annotation theoretically compares cell expression profiles or specific marker genes in cell clusters with annotated databases. Annotation methods are mainly divided into 3 categories, namely marker gene database-based, correlation-based and annotation by supervised classification. SingleR^64^ is a commonly used correlation-based annotation method, which labels cells based on the reference samples with the highest Spearman rank correlations, focusing on the significant distinctions across cell types by using just the marker genes between pairs of labels. Other automated cell-type annotation methods have been evaluated across a wide range of tissues, sample conditions, and applications.^101^ Moreover, although automatic annotation is time-saving and more objective than manual annotation, it still requires supplementation and improvement through manual annotation in many cases.

### 2.7. Gene-level analysis

Differentially expressed genes (DEGs) analysis is a widely used approach to identify genes that exhibit differential expression between populations in both scRNA-seq and bulk RNA-seq experiments. However, the numerous DEG methods available vary greatly in terms of the number and characteristics of DEGs they detect, as well as their stability and potential biases. These factors are compared in this study.^102^

After obtaining the DEGs, the Gene Ontology and Kyoto Encyclopedia of Genes and Genomes databases can be used for term or pathway enrichment analysis. Moreover, gene set enrichment analysis (GSEA) is a supervised method that assesses the statistical significance or concordance of predefined gene sets between two biological states. However, its application is typically limited to case/control experimental designs and requires prior analysis of sample differences.^67^ Superior to GSEA, gene set variation analysis is an unsupervised, nonparametric manner used for analyzing complex and highly heterogeneous pathway activity over a sample population.^68^ Nonetheless, because there are no gold standard expression datasets, the reproducibility and sensitivity of gene set analysis remain to be addressed.^103^

Gene regulatory networks (GRNs) are frequently utilized to illustrate the combinations of active transcription factors that interact with a specific set of cis-regulatory regions in the genome, resulting in a unique gene expression profile for each cellular function or stable state.^104^ A systematic assessment of methods for inferring GRNs has been published.^105^

### 2.8. Cell-level analysis

Pseudotime analysis reveals the regularity of the dynamic process of the cell, and the cells are arranged along the trajectory according to the differentiation expression during the process.^106^ The inferred pseudotime is a 1-dimensional coordinate assigned by trajectory and because all cells were collected at once, the notion of pseudotime does not represent real-time. Existing algorithms for trajectory inference are complementary and show optimal performance based on the characteristics of the data.^107^

Velocity estimation employs the ratio of unsliced and sliced mature mRNA abundance to describe the rate of gene expression changes at a specific time point for individual genes.^76^ This approach overcomes the limitation of pseudotime analysis, which only provides static snapshots of cellular states and fails to capture dynamic changes in scRNA-seq data. Original velocity estimation model^76^ requires a number of constraints such as all genes having the same rate of splicing or the presence of steady states. ScVelo,^75^ however, is a likelihood-based dynamical model that expands RNA velocity estimation to transient and heterogeneous subpopulation kinetics systems.

Cell-cell communication (CCC) mediated by exchanging metabolites and ligand–receptor complexes is crucial to the development, differentiation, and function of cells.^108^ CCC can be inferred from scRNA-seq data at both the individual cell and cell cluster levels using computational methods. However, due to the occurrence of several cell contacts between adjacent cells and the lack of tissue spatial location information in scRNA-seq, integrating it with spatial transcriptome may alleviate limitations in studying CCC.^109^

Compositional analysis requires sufficient cell and sample numbers to estimate cell-type proportion in a sample and evaluate expected background variation.^110^ Because of the limited sample sizes and compositionality of scRNA-seq data, existing models either require advanced cell clustering assignment or cannot avoid false univariate inferences due to negative correlations, do not adequately reflect the overall cell-type composition.^77,111^

## 3. GENERATION OF MM CELL ATLASES

### 3.1. Clonal evolution patterns

MM is a neoplastic disease of plasma cells, characterized by the clonal proliferation of malignant plasma cells in the BM. The disease has a complex evolutionary process resulting from multiple genomic and molecular expression events and cellular heterogeneity in the BM microenvironment (BME). The clinical spectrum of the disease encompasses an asymptomatic stage, such as MGUS with suspected malignant plasma cells producing abnormal monoclonal antibodies (M-protein) in the blood, and a more advanced stage, SMM, characterized by a high proportion of malignant plasma cells in the BM and/or M-protein in the blood.^112^ Individuals with SMM, although asymptomatic, are genetically mature entity whereby molecularly indistinguishable from those with active MM.^113,114^ The genetic events in the progression from asymptomatic to MM can be broadly divided into 2 stages. The primary genetic events begin in a maturing B cell clone and are commonly divided into nonhyperdiploid (non-HRD) and HRD endotypes. The non-HRD tumors harbor immunoglobulin heavy chain (IGH) locus translocations, predominantly involving t(4;14), t(6;14), t(11;14), t(14;16), t(14;20) and deletion of 13q. HRD tumors refer to the presence of trisomy of chromosomes such as 3, 5, 7, 9, 11, 15, 19, and 21.^115^ In accordance with Darwinian evolution theory, genetic alterations that confer a fitness advantage to a clone over other populations are preserved under selective pressure from the tumor microenvironment such as immune surveillance, clonal competition, or drug treatment. Those genetic changes carried by these subclones of cells preserved under selective pressure may be considered driver mutations that play a significant role in tumor clonal evolution. To date, correlative studies have identified more than 80 driver mutations and the genetic landscape has been well established.^116–121^ Some of these mutations are found in a large proportion of patients or affect the pathways, such as KRAS and NRAS mutation in MAPK pathway, TP53, ATM, and ATR mutations in the DNA repair pathway, translocations or copy number variations of MYC, and potential tumor suppressor genes (DIS3 and FAM46C) may be regarded as the independent risk factors of disease progression.^115^

**Figure 2.**
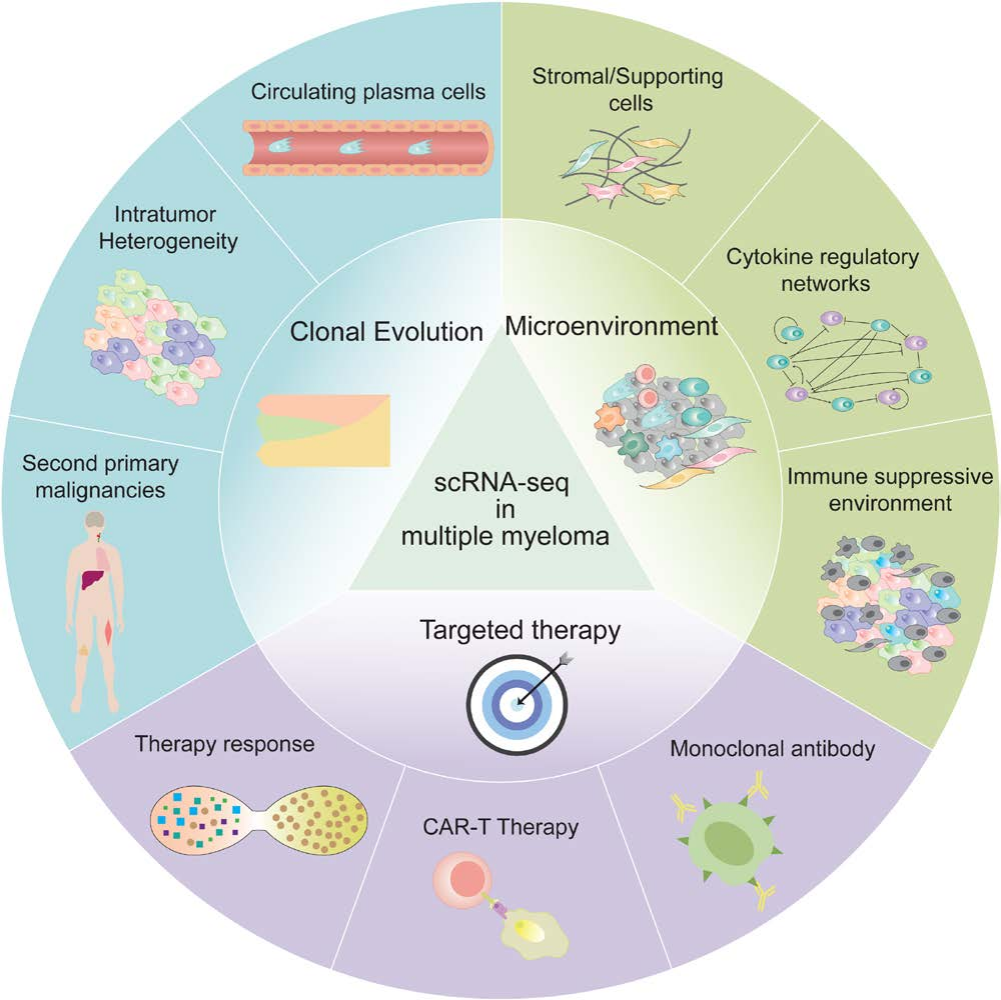
Advances of scRNA-seq in multiple myeloma. To date, single-cell RNA sequencing (scRNA-seq) has been applied to study the clonal evolution, bone marrow microenvironment, and targeted therapy of multiple myeloma.

With the implementation of scRNA-seq techniques, a deeper understanding of the transcriptional heterogeneity among individual cells has been unveiled. The advancements in scRNA-seq research for MM have been summarized in Table 3 and Figure 2. One of the initial studies utilizing multi-omic scRNA-seq and bulk DNA sequencing to examine longitudinal samples from 14 individuals at varying stages of disease, revealing patient-specific plasma cell profiles and immune cell expression variation. Unique subpopulations of plasma cells exhibit transcriptional stability during the progression from asymptomatic SMM to overt disease but demonstrate dynamic emergence or loss during the transition from newly diagnosed MM (NDMM) to RRMM.^131^

**Table 3.** Research progress of single-cell RNA sequencing in MM.

Author	Year	Participants (n)	Cell source	Sample type and total number of single cell (n)	Platforms	Major findings	Ref
Merz et al	2022	10; RRMM (n = 3), NDMM (n = 7)	Bone marrow	PCs (n = 148,630)	10× Genomics Chromium; Illumina NovaSeq 6000	Identified transcriptional changes in PC after therapy; compared with PC from BM, the expression of gene from OL was heterogeneous	^122^
Li et al	2022	13; NDMM (n = 10), healthy donors (n = 3)	Bone marrow, peripheral blood	BM cells (n = 241,440)	10× Genomics Chromium; Illumina NovaSeq 6000	Identified a subset of ZNF683^+^ NK cells with downregulation of activating receptors and upregulation of inhibitory receptors; knockout of ZNF683^+^ in NK cells reversed the exhaustion	^123^
Johnson et al	2022	4; SMM (n = 2), MM (n = 2)	Bone marrow	CD138^+^ cells	10× Genomics Chromium; Illumina NovaSeq 6000	Demonstrated the application of the Diagnostic Evidence GAuge of Single cells (DEGAS) framework; PHF19^high^ myeloma cells are correlated with progression	^124^
Xu et al	2022	1	Bone marrow (ND to PD6); extramedullary plasmacytoma (PD8); peripheral blood (PD9)	CD138^+^ PCs and PBMCs (n = 14,000)	10× Genomics Chromium; Illumina HiSeq X-10	Identified a potential driver gene RUNX3 for RRMM through assessing dynamic genomic changes in temporal consecutive samples	^125^
He et al	2022	18; NDMM (n = 12), RRMM (n = 6)	Bone marrow	CD138^+^ cells	10× Genomics Chromium; Illumina NovaSeq 6000	Identified the relevance of intratumor heterogeneity; discovered novel biomarkers for potential therapy	^126^
Zeng et al	2021	GSE118900 dataset	NA	NA	NA	Constructed 20-genes signature to predict survival; revealed that downregulation of CCND1, upregulation of oncogene FGFR3 and t(4;14) associate with late stage of MM	^4^
Xu et al	2021	17; IgG myeloma (n = 10), other (n = 7)	Bone marrow; extramedullary tissues; tumor-bearing mouse	CD138^+^ cells	NA	Confirmed differential regulation of macrophage MIF expression in MM; suggest a possible pathogenic role of MIF in extramedullary disease transmission	^127^
Waldschmidt et al	2021	4; BRAF-mutated myeloma (n = 3), healthy donors (n = 1)	Bone marrow; peripheral blood (before, after 1 week, at clinical relapse to treatment)	Myeloma cells and normal PCs (n = 1,495)	Smart-seq2; Illumina NextSeq 500	Reveals metabolic reprogramming as a resistance mechanism in BRAF-Mutated multiple myeloma; OxPhos is an energy source for drug-persistent myeloma cells and negatively correlated with MAPK activation	^128^
Tirier et al	2021	20; RRMM (n = 20)	Bone marrow	PCs (n = 83,201); BME cells (n = 129,203)	10× Genomics Chromium; Illumina HiSeq 4000	RRMM cells shape an immune-suppressive BME by accumulation of PD1^+^ γδ T cells and microphages the depletion of hematopoietic progenitors	^129^
Samur et al	2021	1	Bone marrow	CD138^−^ cells and BMMCs (n = 37,656)	10× Genomics Chromium; Illumina	Initial CAR-T cell infusion yielded BCMA clones with biallelic deletion, resulting in a lack of CAR-T cell proliferation after the second infusion	^130^
Liu et al	2021	14	Bone marrow	PCs (n = 17,267); immune cells (n = 57,719)	10× Genomics Chromium; Illumina HiSeq 4000; Illumina NovaSeq 6000	Validated the differential expression of AP-1 complex in plasma cell subsets; downstream targets of AP-1 (IL-6 and IL-1B) may modulate inflammatory responses	^131^
Hogg et al	2021	1; chronic lymphocytic leukemia (n = 1)	Peripheral blood	BMMCs (n = 5240)	NEB E7760; Ilumina NextSeq 500	Identified NCOR1 and HDAC3 transcriptional co-repressors as the antagonists of P300/CBP; the steady-state histone acetylation-methylation balance can control cellular transcription	^132^
Hirabayash et al	2021	1	Bone marrow	PMC (n = 1276); normal BMMCs (n = 86,493)	10× Genomics Chromium; Illumina HiSeq 2500	A3B protein expression is higher in G2/M phase than in G1 or S phases in myeloma	^133^
Goicoechea et al	2021	458	Bone marrow	MRD tumor cells (n = 25,600 median)	10× Genomics Chromium; Ilumina NextSeq 500	Uncovered the selection of MRD clones in high-risk MM; supported undetectable MRD as a potential therapy endpoint	^134^
Frede et al	2021	10; RRMM (n = 8), healthy donors (n = 2)	Bone marrow; peripheral blood	Myeloma cells and CD45^+^ immune cells (n = 6955)	SmartSeq2; Illumina NextSeq 500	Aberrantly activated transcriptional regulators in MM show a loss of lineage restriction; drug treatment can induce the expression of the surface protein CXCL4 and serve as a potential target	^135^
de Jong et al	2021	20; NDMM (n = 13), healthy donors (n = 7), NDMM during treatment (n = 14)	Bone marrow	Nonhematopoietic mononuclear cells (n = 19,983 from MM, n = 7038 from control bone marrow)	10× Genomics Chromium; Illumina NovaSeq 6000	Identified the role of myeloma-specific inflammatory mesenchymal stromal cells in tumor survival and immune modulation; interferon-responsive effector T cell and CD8+ stem cell memory T-cell populations are potential sources of stromal cell–activating cytokines; successful antitumor induction therapy is unable to revert bone marrow inflammation	^136^
Da Via et al	2021	1; enrolled in the KarMMa trial (n = 1)	Bone marrow (before lymphodepletion therapy, infusion of CAR T cells and at the time of progression)	BMMCs	10× Genomics Chromium; Illumina NovaSeq 6000	Identified the relationship between TNFRSF17 (BCMA) homozygous deletion clone and immune escape; heterozygous TNFRSF17 deletion theoretically correlates with BCMA loss after immunotherapy.	^137^
Croucher et al	2021	NA; mice were assigned according to age and M-protein levels	Mouse bone marrow	BM cells (n = 104,880)	10× Genomics Chromium; Illumina HiSeq 2500	A precursor progression model was presented in Vκ*MYC mice; it demonstrates a progressive activation pattern of a subclonal program associated with GCN2 stress response during myeloma progression	^138^
Cohen et al	2021	67; healthy donors (n = 11), NDMM (n = 15), RRMM (n = 41)	Bone marrow	PCs (n = 51,297)	MARS-seq; Illumina NextSeq 500	Identified the relevance of PPIA deletion or inhibition to the sensitivity of MM tumor cells to proteasome inhibitors	^139^
Cho et al	2021	157; NDMM (n = 157)	Bone marrow; peripheral blood	NK cells (n = 5,000)	10× Genomics Chromium; Illumina HiSeq-X	Indicated the role of adaptive NK cells in mediating ADCC in MM; selective use of adaptive NK cells can better predict and improve the therapeutic effect of Daratumumab	^140^
Chen et al	2021	6	Bone marrow (fresh and frozen/thawed cells)	CD138^+^ cells (fresh n = 37,464, frozen n = 49,206); CD138− cells (fresh n = 9,131, frozen n = 21,586)	10× Genomics Chromium; Illumina NovaSeq 6000	Found a better condition for cryopreservation; CD138− cell-type proportions have minimal alterations	^141^
Alameda et al	2021	79; healthy donors (n = 9), light-chain AL (n = 32), MM (n = 32), MGUS (n = 6)	Bone marrow; peripheral blood	PCs (n = 80,239)	NA	Provided a new perspective for normal PC development and transcriptional reorganization in AL and other monoclonal γ diseases.	^142^
Zavidij et al	2020	32; healthy donors (n = 9), MGUS (n = 5), low-risk SMM (n = 3), high-risk SMM (n = 8), MM (n = 7)	Bone marrow	Frozen CD45^+^CD138− cells (n = 4463 from healthy donors, n = 2799 from MGUS, n = 1,782 from SMMl, n = 4,420 from SMMh, n = 5,563 from MM)	10× Genomics Chromium; Illumina HiSeq 2500/4000	Identified the immune changes that play a role in the evolution of premalignant MM	^143^
Xie et al	2020	87; healthy donors (n = 21); NDMM (n = 38), RRMM (n = 28)	Bone marrow; bortezomiBR myeloma cell lines	B cells (from healthy donors); CD138^+^ PCs (from MM)	10× Genomics Chromium; Illumina	Identified an important drug resistance mechanism; combination therapy with SIRT1 inhibitors can make myeloma cells sensitive to proteasome inhibitors	^144^
Ryu et al	2020	15	Bone marrow; extramedullary myeloma; ascites; pleural effusion	Malignant PCs (n = 15,858); BME cells (n = 6782)	10× Genomics Chromium; Illumina HiSeq 2500; Fluidigm C1	Revealed that transcriptional programs associated with the progression of invasive myeloma support autonomous cell proliferation and immune evasion	^145^
Pang et al	2020	5; EMP (n = 5)	Bone marrow; peripheral blood	Circulating PCs; BMMCs	Smart-seq2; Illumina HiSeq 2500	Identified the role of S1PR2 downregulation in initial extramedullary translocation; S1PR2 downregulation promotes cell migration and invasion through NF-κB pathway activation	^146^
Maia et al	2020	1537; prospectively screen (n = 285), larger series (n = 1258)	Bone marrow	CD34^+^ HPCs; BMMCs	Illumina NextSeq 550	Revealed the biological and clinical significance of abnormal hematopoietic function in NDMM; MFC was moderately sensitive for screening abnormal hematopoietic function in NDMM	^147^
Geng et al	2020	21; primarily untreated MM (n = 11), EMP (n = 10)	Bone marrow; peripheral blood	BMMCs (n = 126); circulating PCs	Smart-Seq2; Illumina HiSeq 2500	Identified the upregulation of CXCL12, a chemokine in circulating PC, may be related to the transfer of circulating PC from bone marrow to blood	^148^
Khoo et al	2019	NA	Bone marrow (mice injected with 5TGM1 MM-derived cells)	eGFP+DiD^hi^ dormant cells; eGFP+DiD^neg^ reactivated cells	SMART-seq; MARS-seq; Illumina HiSeq 2500; Illumina Nextseq 500	Dormant MM cells express a distinct transcriptome signature associated with myeloid cell lineage differentiation, cell survival and identifies new treatment targets	^149^
Jang et al	2019	15; MGUS (n = 3), SMM (n = 4), NDMM (n = 5), RRMM (n = 3)	Bone marrow	CD138^+^ cells (n = 597)	Fluidigm C1; Illumina HiSeq 2500	Identified the molecular pathways most significantly affected in MM progression; provided new features for predicting patient outcomes and stratifying treatment	^150^
Bailur et al	2019	33; healthy donors (n = 8), MUGS (n = 14), MM (n = 11)	Bone marrow	BMMC and CD138^−^ cells (n = 9840 from healthy donors, n = 20,286 from MUGS, n = 12,480 from MM)	10× Genomics Chromium; Illumina HiSeq	Described the changes of innate and adaptive immunity in premalignancy; found the mechanism of immune surveillance in myeloma	^151^
Ledergor G et al	2018	40; healthy donors (n = 11), MGUS (n = 7), SMM (n = 6), MM (n = 12), amyloidosis (n = 4)	Bone marrow; peripheral blood	Bone marrow PCs (n = 17,389); CTCs (n = 10,268)	Illumina NextSeq 500	Common malignant pathways consist of unique transcriptional signatures; identified the intratumor heterogeneity of individuals with multiple myeloma; revealed the transcriptional states correlation between CTCs and BM tumor	^113^
Fan et al	2018	NA	Bone marrow; ascites	CD138^+^ cells	Fluidigm C1; Illumina HiSeq 2500	Identified transcriptional signatures relevant to cancer progression through HoneyBADGER; some prominent transcriptional subpopulations were likely driven by alternative, nonclonal mechanisms	^152^
Mitra et al	2016	1; NDMM (n = 1)	HMCLs; bone marrow	HMCLs (n = 528); CD138^+^ (n = 48)	Fluidigm C1; Illumina HiSeq 2500	Developed an R Statistical analysis package and its application into clinically relevant analysis of intratumor heterogeneity; revealed the presence of pre-existing drug-resistant subclones of cells inside untreated myeloma cells.	^153^
Lohr et al	2016	10	Bone marrow; peripheral blood	BMPCs and CTCs (n = 568)	Ion Torrent PGM; Smart-seq2; Illumina NextSeq 500	Demonstrated the clinical potential of a noninvasive biopsy in MM; single CTCs have the same genetic abnormalities as BMPCs; revealed the importance of CTCs analysis in assessment for prognosis	^154^
Melchor et al	2014	6	Bone marrow	CD138^+^ cells (n = 73–243 per individual)	Multiplex qPCR genotyping	Myeloma is composed of 2–6 different major clones; the earliest myeloma-initiating clones only had the t (11;14); clonal diversity and selective pressures are essential foundation for tumor progression and treatment resistance in myeloma	^155^

AL = amyloidosis, BCMA = B-cell maturation antigen, BM = bone marrow, BMMC = bone marrow mononuclear cell, BME = bone marrow microenvironment, BR = b-resistant, CTC = circulating tumor cell, EMP = extramedullary plasmacytoma, HPC = hematopoietic progenitor cell, HMCLs = human myeloma cell lines, MFC = multidimensional flow cytometry, MGUS = monoclonal gammopathy of undetermined significance, MIF = migration inhibitory factor, MM = multiple myeloma, MRD = measurable residual disease, NDMM = newly diagnosed multiple myeloma, PB = peripheral blood, PBMC = peripheral blood mononuclear cell, PC = plasma cell, PD = progressive disease, PMCs = human primary myeloma cells, RRMM = relapsed/refractory multiple myeloma, SMM = smoldering multiple myeloma.

Myeloma progression requires cell-to-cell and cell-to-stroma adhesion, so the appearance of circulating tumor cell (CTC) in the PB at any time during the disease course may indicate relative independence from microenvironmental adhesion and result in extramedullary MM (EMM) through hematogenous spread and unfavorable prognosis.^156–158^ Additionally, the conventional diagnosis of MM involves BM aspiration, which is an invasive and painful procedure that does not facilitate early detection or follow-up monitoring. Lohr et al were the first to employ scRNA-seq to analyze CTCs and MM cells derived from BM, revealing that CTCs harbor identical genetic and transcriptomic information as primary BM cells.^154^ Geng et al and Pang et al proofed that circulating plasma cells (cPCs) abnormally auto-secrete CXCL12 and downregulate the expression of S1PR2 to help extramedullary translocation from the BM.^146,148^

In many cases of MM, the normal balance between bone resorption and new bone formation is disrupted, resulting in bone deterioration and the development of osteolytic lesions.^159^ Osteoclast activation-induced bone damage generally occurs around MM plasma cells rather than in normal BM.^160^ Furthermore, PCs obtained from osteolytic lesions exhibited upregulation of genes associated with myeloma-related bone disease, including DKK1, HGF, and TIMP-1, as well as recurrent downregulation of JUN/FOS, DUSP1, and HBB.^122^

As the survival time of MM patients increases, so does their risk for developing second primary malignancies (SPMs), such as acute myeloid leukemia and myelodysplastic syndromes (MDS).^161–164^ Previous meta-analyses and case reports have identified various risk factors associated with SPMs, whereas scRNA-seq can be utilized to uncover the underlying etiology and molecular mechanisms.

To sum up, based on single-cell analyses, it has been discovered that driver genetic alterations play a crucial role in maintaining the fitness of tumor cells. However, it is also important to note that tumor evolution is also influenced by phenotypic plasticity and interactions that take place within the BME.

### 3.2. Microenvironment

The BME is composed of both cellular and noncellular components. The cellular component contains hematopoietic cells encompassing B cells, T cells, natural killer (NK) cells, osteoclasts, and nonhematopoietic cells such as fibroblasts, osteoblasts, endothelial cells, and mesenchymal stromal cells (MSCs). The extracellular matrix, oxygen concentration, and the liquid milieu (cytokines, growth factors, and chemokines) constitute the noncellular compartment within the BME that is either generated or influenced by the cellular compartment.^165,166^ Bailur et al first applied the scRNA-seq technique to patients with MGUS and MM, showing a gradual increase in terminal effector T cells as the cancer progressed and describing the immune-suppressive phenotype constructed by tumor-infiltrating myeloid cells: reduced expression of CD86, CD155, and c-KIT and increased PD-L1.^[151]^ Zavidij O et al obtained samples from MGUS, low-risk SMM, high-risk SMM, MM and healthy donors and used MACS to isolate CD138^−^ or CD45^+^ cell fractions, demonstrated a significant predominance of NK cells, nonclassical monocytes and macrophages, and T cells in the asymptomatic stages.^143^ Interferon (IFN) type I secreted by MM cells promotes immunosuppression and favors MM growth, and the upregulation of IFN is already detectable at the SMM stage.^143^ Cho et al distinguished 4 clusters of NK cells by DEGs analysis, respectively adaptive NK, terminal NK, CD56^bright^ NK, and NK-HSP. The expression of CD38 in adaptive NK cells was lower than that in conventional NK cells, thus avoiding CD38 monoclonal antibody daratumumab-induced fratricide and increasing daratumumab efficacy by mediating ADCC to kill tumor cells.^140^ Li et al further divided NK cells into 7 subsets through unsupervised clustering, among which ZNF683^+^ NK subset was significantly increased in MM compared with healthy donors. ZNF683^+^ NK cells demonstrate exhaustion phenotypes characterized by higher expression of NK cell inhibitory markers LAG3 and KIR3DL2 and lower expression of cytotoxic gene SH2D1B, granzyme A gene GZMA and granulysin gene GNLY and can be a target for immunotherapy.^123^

In ND/RRMM patients, there is a group of immature BM progenitor cells known as myeloid-derived suppressor cells (MDSCs) that accumulate in both the BM and PB. These MDSCs are highly heterogeneous and have the potential to differentiate into mature and functional osteoclasts.^167–169^ They also can promote MM growth while inducing the generation of Treg cells and suppressing T-cell–mediated immune responses through secreting ARG1, ROS, COX2, iNOS, IL-6, and IL-10 and limiting the extracellular factors required for T-cell activation.^168,170^ There is no relevant study applying scRNA-seq to this highly heterogeneous population of MDSCs. The heterogeneity of MDSCs remains to be explored and can be a potential target for immunotherapy.^171^

Furthermore, it has been demonstrated that the interactions between MM plasma cells and the BME play a critical role in regulating the dormancy of myeloma cells. Dormant myeloma cells may disseminate early in the disease course, acquire resistance to conventional treatments targeting proliferating cells, and persist as minimal residual disease (MRD), which can be reactivated to trigger disease relapse.^172^ Khoo et al identified a transcriptome signature of dormant MM cells in mice, characterized by the upregulation of Irf7, Spic, AXL, FCER1G, CSF1R, SIRPA, and VCAM1 genes.^149^ Further investigation into parallel dormancy genes in clinical samples from healthy donors and MM patients revealed that the proportion of AXL^+^ cells in MGUS patients was higher than that in MM patients, while the number of AXL^+^ cells in patients with recurrent disease was the least. This implies that the inactivation of AXL liberated cells from dormancy and facilitated MM cell proliferation, which may be associated with inferior survival outcomes.

In summary, researchers have used scRNA-seq to categorize immune cells such as NK and T cells into subpopulations with diverse roles at a greater resolution, illustrating the variety of traditional immune cell subgroups. In addition, several studies have shown that cells in the microenvironment promote further tumor progression by interacting with myeloma cells.^159,173–177^ In turn, the myeloma cells can shape a protective immune microenvironment by secreting cytokines to accumulate the regulatory immune cells and cytotoxic but impaired cells and interacting with myeloid components to assist their growth, expansion, and immune escape.^129,145^

### 3.3. Targeted therapy

Over the past few decades, the overall survival rate of MM patients has remarkably improved due to advancements in autologous stem cell transplantation and novel drugs such as proteasome inhibitors (PIs), immunomodulatory drugs, and monoclonal antibodies.^178^ However, given the high heterogeneity of this disease, certain driver genes may be present in some patients but not others.

For KYDAR clinical trial patients or primary refractory MM (PRMM) patients, this study established a unique MM resistance pattern including stimulation of proteasome machinery (PSMB4 and PSMA2), mitochondrial stress (COX6C, COX7A2), ER and UPR pathway (PPIA, STMN1), as well as downregulation of PC checkpoint genes. The presence of this characteristic within a patient cohort is strongly correlated with an unfavorable prognosis. PPIA represents a major hallmark of the resistance signature and an ideal target for drug intervention. The combination of PIs and PPIA inhibitors may enhance the therapeutic efficacy of PIs, resulting in more effective elimination of tumor cells.^139^ From the perspective of transcriptional regulators, in addition to a set of regulations shared between malignant and normal plasma cells (including XBP1, PRDM1, and IRF4), aberrantly activated transcriptional regulators ELF3 and TEAD in MM may indicate widespread alterations in cis-regulatory regions, suggesting a loss of lineage restriction rather than defective differentiation of MM cells. This suggests that drugs can be used to inhibit the aberrant differentiation of MM cells to limit their malignancy. Moreover, therapy can modulate the activation of transcriptional regulators to regulate the expression of downstream genes and cell surface proteins, which may serve as potential targets for immunotherapy.^135^

Furthermore, in recent years, chimeric antigen receptor (CAR) T-cell therapy targeting B-cell maturation antigen (BCMA) has demonstrated rapid and profound responses in treating MM/RRMM. However, long-term maintained remissions are not achieved by most patients and relapse following therapy is frequently observed.^179,180^ The mechanisms of resistance to CAR-T cell therapy mainly include CAR failure (insufficient expansion or cytotoxicity), antigen modulation (downregulation, DNA mutation, or internalization), inhibitory regulation (elevations of CAR-Treg, anti-CAR-T antibody, or inflammatory cytokines).^181–183^ A recent study found that MM relapse following CAR-T therapy may relate to the trogocytosis and internalization or biallelic loss of BCMA.^130,137,184^

Most of the current therapies target either tumor cells or immune cells, but inflammatory MSCs in the BM microenvironment remain unaffected by successful antitumor therapy. This may contribute to disease persistence or relapse and suggests a potential avenue for treating MM.^136^ Finally, MM remains an incurable disease with a large number of relapsed/refractory patients. For patients with MM subsets that are already resistant before treatment, treatment will remove the inhibition of sensitive population, thus speeding up the growth of the resistant population, which may eventually worsen the disease.^185^ Therefore, the development of personalized targeted drugs and identification of potential individualized therapeutic targets are crucial in the management of MM.

## 4. CONCLUSION

Since its inception in 2009, scRNA-seq has revolutionized our understanding of the intricate and diverse RNA expression patterns within individual cells. As technological advancements in cell capture techniques, sequencing throughput, and computational algorithms progress, we are gaining unprecedented insight into the complex biological mechanisms underlying cell-type composition, GRNs, and cellular differentiation dynamics. With each new development, we are able to unlock increasingly intricate details about the fundamental biology of life at the cellular level. Numerous groups have applied scRNA-seq to the MM, it has been crucial in identifying new immune cell subsets, elucidating the degree of cellular malignancy in myeloma patients at different stages, and searching for mechanisms of drug resistance and relapse after treatment. After successful antitumor therapy, although myeloma cells have been effectively controlled, the presence of MRD and an inflammatory microenvironment in the BM may still predispose to disease recurrence. Our study covers the scRNA-seq platforms and analytic tools, which may be utilized to build a systematic research pipeline for future investigations. Simultaneously, we describe the key findings of prior scRNA-seq applications in MM, which may be utilized as a reference for prospective study.

## AUTHOR CONTRIBUTIONS

J.D. conceived the idea for the review article. X.R.G. was involved in writing—original draft preparation and figures. All authors were involved in writing—reviewing and editing.

## ACKNOWLEDGMENT

This work was supported by the Shanghai Shenkang Hospital Development Center (No. SHDC2020CR2070B).

The authors thank Jian Hou and Yangjia Cao for proofreading the manuscript. And the Figure was partly generated using Servier Medical Art, provided by Servier, licensed under a Creative Commons Attribution 3.0 unported license.
